# The Impact of Vitamin D on the Immunopathophysiology, Disease Activity, and Extra-Musculoskeletal Manifestations of Systemic Lupus Erythematosus

**DOI:** 10.3390/ijms19082355

**Published:** 2018-08-10

**Authors:** Anselm Mak

**Affiliations:** 1 Department of Medicine, National University of Singapore, Singapore 119228, Singapore; mdcam@nus.edu.sg; 2 Division of Rheumatology, University Medicine Cluster, National University Health System, Singapore 119228, Singapore

**Keywords:** lupus, systemic lupus erythematosus, vitamin D, disease damage, disease activity

## Abstract

Over the past two decades it has been increasingly recognized that vitamin D, aside from its crucial involvement in calcium and phosphate homeostasis and the dynamics of the musculoskeletal system, exerts its influential impact on the immune system. The mechanistic roles that vitamin D plays regarding immune activation for combating infection, as well as pathologically and mediating autoimmune conditions, have been progressively unraveled. In vitro and in vivo models have demonstrated that the action of vitamin D on various immunocytes is not unidirectional. Rather, how vitamin D affects immunocyte functions depends on the context of the immune response, in the way that its suppressive or stimulatory action offers physiologically appropriate and immunologically advantageous outcomes. In this review, the relationship between various aspects of vitamin D, starting from its adequacy in circulation to its immunological functions, as well as its autoimmune conditions, in particular systemic lupus erythematosus (SLE), a prototype autoimmune condition characterized by immune-complex mediated inflammation, will be discussed. Concurring with other groups of investigators, our group found that vitamin D deficiency is highly prevalent in patients with SLE. Furthermore, the circulating vitamin D levels appear to be correlated with a higher disease activity of SLE as well as extra-musculoskeletal complications of SLE such as fatigue, cardiovascular risk, and cognitive impairment.

## 1. Vitamin D, Its Nature and Impact on Various Body Systems

### 1.1. The Physiology and Health Impact of Vitamin D

Vitamin D is one of the steroid-based vitamins that chiefly regulates the absorption of calcium from the intestine, facilitates calcium reabsorption in the kidneys, and mobilizes calcium and phosphate from bones by activating the osteoclasts [1,2]. Humans obtain vitamin D from two sources, a small amount from diet, while the majority is synthesized in the epidermal layer of the skin by converting 7-dehydrocholesterol in the epidermis to cholecalciferol (vitamin D_3_) via the action of ultraviolet B between the wavelengths of 280 and 315 nm [3]. By binding to the vitamin D-binding protein in the bloodstream, cholecalciferol is transported to the liver, where it is hydroxylated to 25-hydroxycholecalciferol [25-(OH)D_3_] under the action of hepatic 25α-hydroxylase [4]. Subsequently, in the final processing step, 25-(OH)D_3_ is converted to 1-25α dihydroxycholecalciferol (1,25-[OH]_2_D_3_) or calcitriol, through the action of 1α-hydroxylase in the renal cortices [4]. Unlike the 1α-hydroxylation in the liver, the 25α-hydroxylation in the kidneys is a tightly regulated process under the meticulous control of the parathyroid hormone (PTH) and serum calcium and phosphate levels [5]. Furthermore, as a checking mechanism to prevent the overproduction of 1,25-(OH)_2_D_3_, which can otherwise induce hypercalcaemia and hyperphosphataemia, 1,25-(OH)_2_D_3_ is capable of stimulating the 24α-hydroxylation of 25-(OH)D_3_, leading to the formation of the physiologically-inactive 24, 25-(OH)_2_D_3_ [6]. Being an active form of vitamin D, 1,25-(OH)_2_D_3_ initiates the downstream signaling pathways with its association to the vitamin D receptors (VDR), a group of nuclear receptors that heterodimerize with the retinoid X receptor (RXR) and bind to target DNA sequences, named the vitamin D responsive elements (VDRE). The VDRE is situated physically and/or functionally to the promotor region of the target genes that functions to modulate the cell growth, proliferation, and apoptosis, amongst other physiological functions [7].

Besides maintaining calcium and phosphate homeostasis, vitamin D has been demonstrated to diversely impact several body systems, as evidenced by the observation that its deficiency can lead to general health issues [3,8]. For instance, a low vitamin D level has been shown to be related to an increased prevalence of hypertension; cardiovascular disease; respiratory infections; periodontal disease; certain malignancies including breast, colon, and prostate cancers [3,9,10,11]; and autoimmune conditions such as multiple sclerosis (MS), type 1 diabetes mellitus (DM), rheumatoid arthritis (RA), and systemic lupus erythematosus (SLE) [12,13,14]. Such observations have led to the postulation that vitamin D supplementation may be a favorable strategy to combat certain health issues, apart from those related to the musculoskeletal system and calcium-phosphate homeostasis. In fact, it is evident that adequate vitamin D levels help reduce the fall risk and the prevalence of certain types of cancers including breast cancer [15], as well as that of autoimmune conditions, such as RA and MS, in a dose-dependent fashion [16,17,18]. Besides the potential reduction in disease prevalence, adequate vitamin D also exerts an impact on survival. A systematic review and meta-analysis revealed that an increase in the serum 25-(OH)D_3_ level from 54 to 110 nmol/L was associated with an estimated reduction of mortality by 20% in patients with vitamin-D sensitive diseases, including cardiovascular disease, cancers, respiratory infections, DM, Alzheimer’s disease, falls, and MS [19]. In addition, an increment of the serum vitamin D level to 105 nmol/L was shown to reduce the incidence of cardiovascular disease and cancers, including colorectal and breast malignancy, by 15% and 30%, respectively [3]. Nevertheless, the results of the studies addressing the potential relationship between the amount of vitamin D intake or the vitamin D levels and the prevalence of various vitamin D-related disease conditions should be interpreted with caution, because issues such as the difference in study design; insufficient sample size and statistical power; heterogeneity in meta-analyses; and intangible confounding factors, including assessment of sun exposure, skin pigmentation, drug interactions, and alcohol use, may confound accurate interpretation. 

### 1.2. Vitamin D and the Immune Cells

#### A Brief Outlook

Besides the kidneys, immune cells, including the dendritic cells (DC), macrophages, monocytes, and lymphocytes (B and T cells), are capable of converting 25-(OH)D_3_ to 1,25-(OH)_2_D_3_ [20,21,22,23]. Such a local regulation of the intracellular active vitamin D level theoretically endows the immunocytes with a higher level of vitamin D-related immune response control in the inflamed sites, and it also directly demonstrates the important role that vitamin D contributes to the immune system. VDR is also expressed in immunocytes, and hence, the immune-physiological effects of 1,25-(OH)_2_D_3_ in various immunocytes can be implemented via binding with the VDR, which mediates the gene transcription via association with the VDRE [7]. Another piece of evidence that suggests a potential role of vitamin D in the immune system and the mediation of autoimmune diseases is that a few well-described VDR polymorphisms, namely, *ApaI*, *FokI*, *BsmI*, and *TaqI*, were demonstrated to be associated with the risk of the development of autoimmunity [24,25]. Nevertheless, data regarding the mechanism as to how VDRE affects the downstream functions of immunocytes are limited. With the use of the chromatin precipitation technique, followed by deep sequencing datasets of VDR-binding sites from calcitriol-treated human B cells and monocytes, the transcriptome data identified a small set of VDR targeted genes that were upregulated [26]. The upregulated calcitriol-responsive VDR target genes demonstrated significant relationships with leukocyte transendothelial migration, Fcγ receptor-mediated phagocytosis, and transcriptional regulation by VDR [26], illustrating a pivotal role of the VDR target genes in immune regulation [26]. In addition, in the FoxP3^+^ regulatory T cells (Treg), 1,25-(OH)_2_D_3_-associated VDR was shown to target the VDRE, which comprises an intronic conserved noncoding sequence region of the human *FoxP3* gene, leading to the enhancement of the FoxP3 promotor activity as well as the resultant suppressive activity of Treg, secondary to the vitamin D treatment [27].

### 1.3. Action of Vitamin D in Immunocytes of the Innate Immune System

#### 1.3.1. Dendritic Cells

Immunocytes, ranging from the DC to lymphocytes, have been shown to be influenced by vitamin D because of their expression of VDR [7,23]. During the early differentiation stage, the DC that was differentiated from monocytes in the presence of 1,25-(OH)_2_D_3_ remained in a tolerogenic state, as characterized by a reduction in interleukin (IL)-12 and an increase in IL-10 production, leading to the subsequent reduction in allogenic T-cell activation and enhanced Treg differentiation [28]. However, the effect of 1,25-(OH)_2_D_3_ on inflammatory DCs, which are differentiated and matured inflammatory, are much less substantial [28]. While the role of vitamin D in lupus DC has not been well investigated, being the main driver of interferon (IFN)α production and antigenic presentation, by plasmacytoid DC and myeloid DC, respectfully, the potential role of vitamin D in lupus DC warrants further investigation.

#### 1.3.2. Macrophages

Unlike the unidirectional action of 1,25-(OH)_2_D_3_ in DC, 1,25-(OH)_2_D_3_ has dual effects in the macrophages, depending on their activation status. Taking bacterial infection as an example, during the initial phase of infection, 1,25-(OH)_2_D_3_ enhances the differentiation of monocytes to macrophages [29]. In tuberculosis infection, where the macrophage IFNγ receptors are stimulated, the resultant activation of Cyp27Bq potentiates the conversion of 25-(OH)D_3_ to 1,25-(OH)_2_D_3_ by 1α-hydroxylation in the macrophages [30]. 1,25-(OH)_2_D_3_ induces the production of IL-1β that enhances the production of the antimicrobial cathelicidin, attempting to facilitate the clearance of the pathogens by the macrophages [31]. At the later phase of infection, where the macrophages are sufficiently or over activated, 1,25-(OH)_2_D_3_ acts to dampen the proinflammatory response by reducing the production of IL-1β, IL-6, tumor necrosis factor (TNF)α, nuclear factor kappa-B ligand (RANKL), nitric oxide, and co-oxygenase-2 (COX-2), and increasing the production of IL-10, which is an anti-inflammatory cytokine [32,33]. While data regarding the action of vitamin D in lupus macrophages are scant, such dual effects impacted by vitamin D on macrophages would be potentially operative in the macrophages from patients with SLE, as lupus macrophages have also been documented to possess dual inflammatory and anti-inflammatory properties [34,35].

### 1.4. Action of Vitamin D in Immune Cells of Adaptive Immune System

Upon activation, different T cell subsets, including the CD4^+^, CD8^+^, and TCRγδ^+^ T cells, express VDR [36]. The subsequent T-cell activation, which involves the propagation of T-cell receptor (TCR) signaling, has been shown to require an activation of VDR via its association with 1,25-(OH)_2_D_3_ [37]. Compared with DC and the B cells (which will be discussed in a subsequent section), the actions of vitamin D on T cells are more complicated, because of the differential actions of vitamin D in different well-described T cell subsets, including CD4^+^ (Th1, Th2, Th17, and Treg) and CD8^+^ T cells. As a result, the impact of vitamin D on T cell physiology is subset-dependent.

#### 1.4.1. CD4^+^ T cells—The Th1, Th2, Th17, and Treg Subtypes

The pathogenic role of the CD4^+^ Th1 T cells in mediating the pro-inflammatory response in SLE has been well described in the literature and extensively reviewed elsewhere [38]. Although insufficient data have yet been shown regarding how vitamin D affects the lupus CD4^+^ Th1 cells, the data thus far have shown that the VDR expression in non-lupus CD4^+^ T cells is not as substantial as other T cell subtypes. Nevertheless, during the initial phase of CD4^+^ T cell differentiation, 1,25-(OH)_2_D_3_ treatment is capable of inhibiting the IFNγ production chiefly via the downregulation of IL-2 production in Th1 cells [39]. However, the role played by vitamin D in inflammatory response and autoimmunity appears to be more substantial in Th2 and Th17 than in Th1 cells, partly due to the low expression of VDR in the latter [40,41]. Th2 cells are able to suppress experimental autoimmune encephalitis (EAE), a murine model of MS that is predominantly Th17 mediated. Upon activation by 1,25-(OH)_2_D_3_, the Th2 cells suppress the Th17-induced inflammatory response in EAE through the transcription factor, GATA3, and the signal transducer and activator of the transcription protein (STAT)-6 activation, although the presence of IL-4 is essential [42]. Similar to MS, Th17 also plays a pivotal role in the pathophysiology of RA. The culture of the peripheral blood mononuclear cells (PBMC), from RA patients with 1,25-(OH)_2_D_3_, has been shown to restore IL-4 levels and Th2 polarization [43], potentially leading to less severe arthritis. Notably, in conditions where IL-4 is abundant, 1,25-(OH)_2_D_3_ does not appear to increase the IL-4 production further, and suppresses other Th subtypes [43]. Collectively, these data demonstrate the potential of the role of vitamin D in Th2 cells for the suppression of Th17-mediated autoimmune disease via GATA3 and STAT-6 activation and IL-4 production, at least in the Th17-driven EAE model.

The Th17 cells are increasingly recognized as playing essential roles in driving a number of autoimmune conditions, including SLE, RA, and MS [43,44,45]. In a murine model of retinal autoimmunity, 1,25-(OH)_2_D_3_ was shown to inhibit Th17 activity and reduce the expression of IL-17 driven cytokines, namely IL-17 and IL-22/23, in CD4^+^ memory and CCR6^+^ T cells [46]. Besides the inhibition of Th17 activity, 1,25-(OH)_2_D_3_ also inhibits Th17 differentiation, as evidenced by the observation that when naïve T cells were given conditions towards Th17 polarization, the presence of 1,25-(OH)_2_D_3_ inhibits the Th17-related cytokines and intracellular transcription factors, including RORC and CCR6 [47]. In a six-month prospective study of 20 lupus patients with vitamin D deficiency, the adequate supplementation of vitamin D (cholecalciferol) led to reductions in the frequencies of Th1 and Th17 CD4^+^ T cells, while the frequency of the Treg cells increased [48]. The SLE disease activity in these patients remained stable, without the necessity to escalate the immunosuppressive therapy [48]. While the mechanism as to how the differentiation and activity of Th17 are affected by 1,25-(OH)_2_D_3_ is not completely understood, current data suggest that the regulation of IL-17A can be mediated by the binding of VDR to the *IL-17A* promotor region, leading to competition with nuclear factor of activated T cells (NFAT) binding in the same promotor site, and the subsequent recruitment of histone deacetylase (HDAC) and RUNX1, which depress the expression of the *IL-17A* gene [49].

One interesting phenomenon with regard to Th17 cells is their plasticity. Upon stimulation with IL-12 and TNFα, Th17 cells manifest Th1 properties by expressing Tbet and IFNγ, and these non-classic Th17 cells appear to be more pathogenic than the classic Th17 cells in driving autoimmunity [50]. Indeed, 1,25-(OH)_2_D_3_ inhibits the proportion of non-classic Th17 cells that express IFNγ and IL-17 [50]. In our preliminary knockout mouse study, we were able to demonstrate the plasticity between the Th1 and Th17 cells, and the superior pathogenic potential of Th17 cells in SLE [51] (which will be discussed in a subsequent section).

Treg cells play a crucial role in dampening the proinflammatory responses in many autoimmune conditions, including SLE, type I DM, RA, and MS [52,53]. In the EAE model, 1,25-(OH)_2_D_3_ induces the expression of FoxP3 in the lymphoid organs, which is IL-10 signaling dependent [54]. The in vitro treatment of Treg with 1,25-(OH)_2_D_3_ induces the production of the IL-10 and expressions of co-inhibitory molecules, including PD1 and CTLA4, which dampen the excessive pro-inflammatory T cell response [55]. Similar to the action of vitamin D in the promotor regions of the Th17 cells, VDR binds to three VDRE regions in the non-coding sequence of the FoxP3 promotor that controls FoxP3 expression in the Treg population [56]. Furthermore, 1,25-(OH)_2_D_3_ enhances the Treg suppressive activity and number by inducing the indoleamine 2,3-dioxygenase (IDO) expression, and yet, low vitamin D levels in the SLE patients dampen the Treg migratory ability [56]. Summarizing the current data described, 1,25-(OH)_2_D_3_ furnishes the suppressive activity on the CD4^+^ T cells, particularly in the Th17 and Th1 subsets that predominantly produce the pro-inflammatory IFNγ and TNFα. In contrast, 1,25-(OH)_2_D_3_ enhances the activity of Th2 and Treg, which chiefly express IL-4 and IL-10 as well as co-inhibitory molecules, including cytotoxic T-lymphocyte–associated antigen 4 (CTLA-4) and programmed cell death-1 (PD1), which dampen the pro-inflammatory responses. As such, 1,25-(OH)_2_D_3_ likely possesses a strong potential to manipulate the immune system against autoimmunity.

#### 1.4.2. CD8^+^ T Cells

The exact pathophysiological function of the CD8^+^ T cells in SLE has not been fully characterized, although studies have demonstrated its potential suppressive action in SLE [38]. While limited data has been shown in lupus CD8^+^ T cells in relation to vitamin D, the current data have demonstrated that CD8^+^ T cells express a higher VDR level than CD4^+^ T cells in the non-SLE setting [36]. An adoptive transfer of VDR^−/−^ CD8^+^ T cells in Rag^−/−^ mice led to severe colitis (predominantly CD8^+^ T-cell mediated), with an increase in the IFNγ and IL-17 expression in the intestine, particularly when IL-10 is absent [57]. Upon 1,25-(OH)_2_D_3_ treatment, the number of hyperactivated CD8^+^ cells were substantially reduced [57]. As for the clinical studies, 1,25-(OH)_2_D_3_ was found to inhibit CD8^+^ activated IFNγ and TNFα secretion in the patients with MS. In the patients with psoriasis, which is a CD8^+^ dominant T-cell mediated autoimmune condition that manifests as chronic cutaneous and joint inflammation, topical vitamin D (calcipotriol) is one of the therapeutic options of the condition [58].

#### 1.4.3. B Cells

B cells are pathologically important in SLE as they produce lupus-related autoantibodies and function as antigen presenting cells (APC) [59]. The role of vitamin D that is played specifically by lupus B cells has only been very scarcely reported. In a cross-sectional study of 32 patients with SLE, the patients with a high B cell activation status were shown to have a lower mean 25(OH)-vitamin D levels compared to those with a low B cell activation [60]. Nevertheless, the involvement of vitamin D in B cells regarding the physiological control of inflammatory responses is evidenced by the knowledge that the VDR associates with VDRE in lymphoblastoid B cell lines [61]. Thus far, the data have shown that the action of 1,25-(OH)_2_D_3_ appears to be dual, depending on the stage of differentiation of the B cells. Studies indicated that 1,25-(OH)_2_D_3_ decreased the proliferation of B cells and immunoglobulin (Ig) class switching, and induced apoptosis [62]. Vitamin D inhibits B cell differentiation by interfering nuclear NF-κB translocation and CD40 co-stimulation [63]. At the other end of the B-cell development spectrum, 1,25-(OH)_2_D_3_ stimulates the development of plasma cells from terminally differentiated B cells, and enhances the migration of plasma cells towards inflammatory mucosal surfaces via the induction of CCR10 receptors [64]. While vitamin D has been shown to reduce the production of lupus-related autoantibodies, such as anti-nuclear antibody (ANA), independent of its impact on B cell differentiation [65,66], VDR binds to the IL-10 promotor and enhances IL-10 production, potentially alleviating autoimmunity [67]. The role that vitamin D exerts on B cells as APC is not clear, although there is one study that demonstrated that CD86 expression was reduced on B cells primed with 1,25-(OH)_2_D_3_, reducing the potency of allogenic T cell stimulation and the subsequent T-cell mediated inflammatory response [68].

## 2. Hypovitaminosis D and Its Impact on Disease Activity and Major Extra-Skeletal Manifestations in Patients with Systemic Lupus Erythematosus

### 2.1. Pathology Related to T Cell Subsets in SLE—A Brief Discussion

SLE is a multi-systemic autoimmune condition characterized by immune-complex induced inflammation as a result of the association between autoantigens and autoantibodies [59]. Upstream to auto-antibody formation, the SLE have been found to be substantially T-cell driven, particularly the CD4^+^ subset [38]. The Th1 and Th17 CD4^+^ T cells are chiefly responsible for driving the pro-inflammatory response in SLE [38]. Using a CD137 ligand (CD137L) knockout B6.MRL.lpr^−/−^ spontaneous murine SLE model, our group has recently demonstrated that while the CD4^+^Tbet^+^ (Th1) subset is increased in the B6.MRL.lpr^−/−^ mice, the absence of the co-stimulatory molecule CD137L increases the severity of SLE via two pathways [50]. Firstly, the absence of a costimulatory function shifts the plastic Th1 subset into the more pathogenic CD4^+^RoRγt^+^ (Th17) T cells. Secondly, the lack of CD137L signaling leads to a reduced serum level and intracellular IL-10 expression secondary to the significant reduction of the splenic CD11b^+^ population [51]. As discussed previously, as 1,25-(OH)_2_D_3_ dampens the proinflammatory response of Th17, it is theoretically possible to suppress the SLE disease with vitamin D treatment. Indeed, at least three clinical studies have demonstrated the beneficial effects of vitamin D in terms of the reduction in the frequency of, as well as the IL-17 expression of, the Th17 cells [46,69,70]. Although these studies were probably not powered enough to demonstrate the clinical improvement of SLE, further experiments with larger sample sizes will be necessary to explore the potential clinical benefit of vitamin D supplementation in Th17 activity. 

### 2.2. Low Vitamin D Level and SLE Disease Activity

As exposure to the UV light is one of the most potent contributors to SLE flare, clinicians frequently advise SLE patients to minimize sunlight exposure and use sunscreen. The effect of drugs such as glucocorticoids, calcineurin inhibitors, and anticonvulsants, as well as compromised 1α-hydroxylation of 25-(OH)D_3_ due to renal insufficiency, further contribute to low vitamin D in patients with SLE [71,72,73].

Since four decades ago, the relationship between vitamin D and SLE has been described, with the first report published in the late 1970s, which described that 7 out of 12 paediatric SLE patients had low serum 1, 25-(OH)_2_D_3_ levels [74]. Thereafter, a number of larger-scale case-control studies confirmed the relationship between hypovitaminosis D and the prevalence of SLE [75,76,77,78], particularly in patients with lupus nephritis [75]. In our recent age- and gender-matched case-control study of 61 SLE patients and 61 healthy controls, we found that a deficiency of the total 25(OH)D_3_ level, as measured by liquid chromatography-tandem mass spectrometry, was significantly more prevalent in the patient group (19.7% vs. 3.3%, *p* = 0.003) [79]. Recently, a meta-analysis of 18 studies consisting of 1083 patients with SLE and 1273 healthy controls showed that the SLE patients had a significantly lower level of vitamin D compared with the healthy controls [76]. In addition, vitamin D deficiency was significantly more prevalent in the SLE patients when compared with the healthy controls, with a relative risk of 2.3 (*p* = 0.002) [80].

To further to the observation that hypovitaminosis D is more prevalent in SLE patients, low vitamin D levels also appear to be associated with a high disease activity of SLE [81,82,83,84,85]. In an observational study comprising 142 patients with 25-(OH)D_3_ level less than 30 ng/mL, the serum level of 25-(OH)D_3_ was found to be significantly higher in those patients with low disease activity (SLE disease activity index [SLEDAI] 1–5), and lower in those with high disease activity [81]. In addition, higher anti-dsDNA levels were found to be associated with low vitamin D levels in SLE patients [60,83]. Furthermore, the relationship between hypovitaminosis D and the SLE disease activity does not spare the paediatric population. In a case-control study of 35 paediatic-onset SLE patients in Taiwan, low serum 25-(OH)D_3_ levels were significantly associated with active SLE disease activity compared to patients with inactive lupus [86]. Moreover, patients with active lupus nephritis had a significantly lower 25-(OH)D_3_ level compared with those without nephritis [86].

## 3. Vitamin D and Its Major Extra-Musculoskeletal Impacts

### 3.1. Cardiovascular Risk and Its Biomarkers

The patients with low vitamin D levels or with a vitamin D deficiency were found to have a high prevalence of cardiovascular risk factors such as dyslipidaemia [87], hypertension [88], fasting glucose and insulin resistance [89], metabolic syndrome [90], positive antiphospholipid antibodies [87], and a high increased hsCRP level [91]. In a prospective study of 890 patients with SLE in a large international inception cohort, multiple logistic regression analyses revealed that patients at the high quantiles of 25-(OH)D were less likely to possess cardiovascular risk factors, including hypertension and hyperlipidaemia, while a non-significant trend of reduction of the hazard ratio of cardiovascular events was noted across successively higher quantiles of 25-(OH)D levels [88].

Recently, biophysical markers of cardiovascular disease such as carotid plaques, carotid intima-media thickness, endothelium-dependent dilation, and arterial stiffness have emerged as potential biomarkers of cardiovascular disease [92]. A few studies have addressed the potential relationship between hypovitaminosis D and the unfavorable alterations of these biophysical cardiovascular risk markers. By measuring the total plaque area (TPA) with the use of carotid ultrasound in a matched case-control study, the 25-(OH)D level was inversely associated with age-adjusted TPA [93]. In addition, a logistic regression model consisting of ACE inhibitor non-use, the 25-(OH)D level and LDL-c levels had a diagnostic accuracy of 84% in predicting the accelerated atherosclerosis [93]. Again, because of the difference in research methodology, the patient characteristics and the operator-dependent nature of the measurement of biophysical markers, not all of these studies detected a significant relationship between vitamin D deficiency and the presence cardiovascular risk factor [94].

### 3.2. Fatigue and Sleep

Fatigue is very prevalent in patients with SLE. Up to 80% of patients with SLE report symptoms of fatigue during their disease course [95]. Fatigue in SLE patients has been found to be related to low serum vitamin D levels [95,96,97,98,99]. A cross-sectional study of 90 patients with SLE found that those with vitamin D deficiency (25-(OH)D_3_ ≤10 ng/mL) reported higher fatigue scores that those with adequate vitamin D levels [100]. In an observational study of 60 patients who took vitamin D_3_, an inverse relationship between the 25-(OH)_3_ level and fatigue score was identified [101]. Apart from fatigue, the vitamin D level was found to be related to sleep quality in the patients with SLE [102]. With the use of the Pittsburgh Sleep Quality Index, a retrospective study of 60 SLE patients revealed a significant relationship between a low serum vitamin D level and poor sleep quality [102].

### 3.3. Cognitive Impairment

While the association between cognitive impairment and low vitamin D has been extensively reported in the general population, especially amongst the elderly, the relationship between cognition and vitamin D in SLE has been scarcely reported. In our recent age- and gender-matched case-control study of 122 subjects, we found that the 25(OH)D_3_ level was significantly lower in the patients with SLE compared with that of healthy individuals [79]. Specifically, the deficiency of 25(OH)D_3_ was associated with a poorer subclinical cognitive function in terms of the total throughput score with the use of the Automated Neuropsychological Assessment Matrix, even after adjusting for age, education, duration of SLE, cumulative steroid dose, SLE disease activity, and SLE-related damaged and anxiety level in patients with SLE [79].

## 4. Vitamin D Supplementation in SLE

Over the past 10 years or so, a few studies have been designed to study the effect of vitamin D supplementation in patients with SLE [7,101,103,104,105]. While some studies demonstrated a reduction of SLE disease activity with vitamin D supplementation coupled with reduction in autoantibody levels [106,107], some did not [100,105]. For instance, in a recently published randomized control trial of vitamin D supplementation (50,000 U weekly for three weeks, then monthly for three months, versus placebo) in 90 patients with SLE, no change was detected in SLE disease activity between the two groups, using the SLEDAI [108]. In one of the largest single-arm prospective observational studies of 1006 patients with SLE with a low 25-(OH)D level (<40 ng/mL) who received vitamin D2 treatment (50,000 units weekly), a 20-unit increase in serum 25-(OH)D level was shown to be associated with a mean reduction of 0.22 points in the SLEDAI scale, corresponding to a reduction of 21% in the odds of active SLE with SLEDAI ≥5 [109]. In the most updated meta-analysis of the three studies, which involved 233 patients in the vitamin D treatment group and 128 in the placebo group, vitamin D supplementation was found to be significantly associated with a reduction in anti-dsDNA positivity (*p* = 0.005), without statistically significant heterogeneity amongst the studies [110]. Furthermore, the vitamin D supplement led to a reduction in fatigue [98,104], decrease in the Th1/Th17 and memory B cells that would otherwise enhance lupus-related inflammation, and increase in Treg cells that dampen lupus-related proinflammatory response [48,104]. While the presence of the type 1-IFN signature has recently been implicated in active SLE, vitamin D supplementation however failed to demonstrate a significant change in the IFN signature after 12-weeks of treatment [105]. As for cardiovascular risk, cholecalciferol supplementation in the patients deficient of vitamin D appears to improve the endothelial function, and calcitriol enhances the endothelial nitric oxide expression in human endothelial cells [111]. Nevertheless, at the time of writing of this review, there is yet to be any published study that demonstrates the clinical benefit of vitamin D supplementation in reducing cardiovascular events and mortality in SLE patients.

## 5. Conclusions

**Figure 1:**
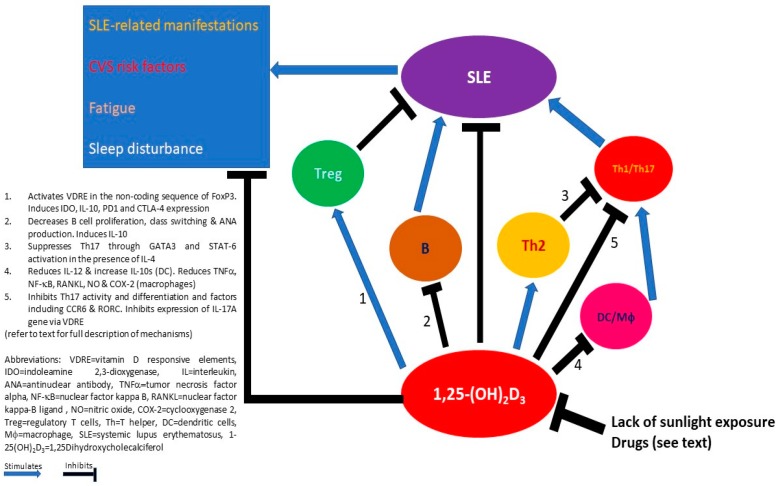
Potential mechanisms of how 1,25-(OH)_2_D_3_ interacts with the environment and immune cells in mediating the clinical manifestations of systemic lupus erythematosus (SLE).

Besides its important involvement in calcium and phosphate homeostasis and the dynamics of the musculoskeletal system, vitamin D has an influential impact on the immune system. Current data have shown that while vitamin D generally suppresses the proinflammatory properties of APC, Th1 and Th17 CD4^+^ T cells, and B cells, it enhances the anti-inflammatory characteristics of Treg and Th2 cells in many autoimmune conditions. While how vitamin D impacts SLE immunocytes needs to be answered by further research, the potentially relevant effects of vitamin D in individual immunocytes, and their cross-talks in the settling of lupus has been proposed (Figure 1). The heightened prevalence of extra-musculoskeletal complications, such as cardiovascular risk, fatigue, sleep disturbance, and cognitive impairment, highlights the potential of vitamin D supplementation as an adjunct therapeutic option for patients with SLE. Although not all clinical trials demonstrated clinically significant benefits of vitamin D supplementation in patients with SLE, in terms of an improvement of SLE disease activity and its associated complications, the theoretical clinical advantage of an adequate vitamin D level, the positive signals detected in various meta-analyses, and the favorable tolerability of vitamin D supplementation in nearly all of these clinical trials warrant sufficient vitamin D intake, supplementation, and monitoring in all patients with SLE in current clinical practice.
